# Supplementary material for: Transcriptome Analysis Reveals the Defense Mechanism of Cotton against Verticillium dahliae Induced by Hypovirulent Fungus Gibellulopsis nigrescens CEF08111
Source: Int J Mol Sci. 2023 Jan 12;24(2):1480. doi: 10.3390/ijms24021480 (PMC9863408; doi:10.3390/ijms24021480)
Supplement: Supplementary file 1 [file ijms-24-01480-s001.zip › Supplementary Figures S1-S12.pdf]

Figure S1

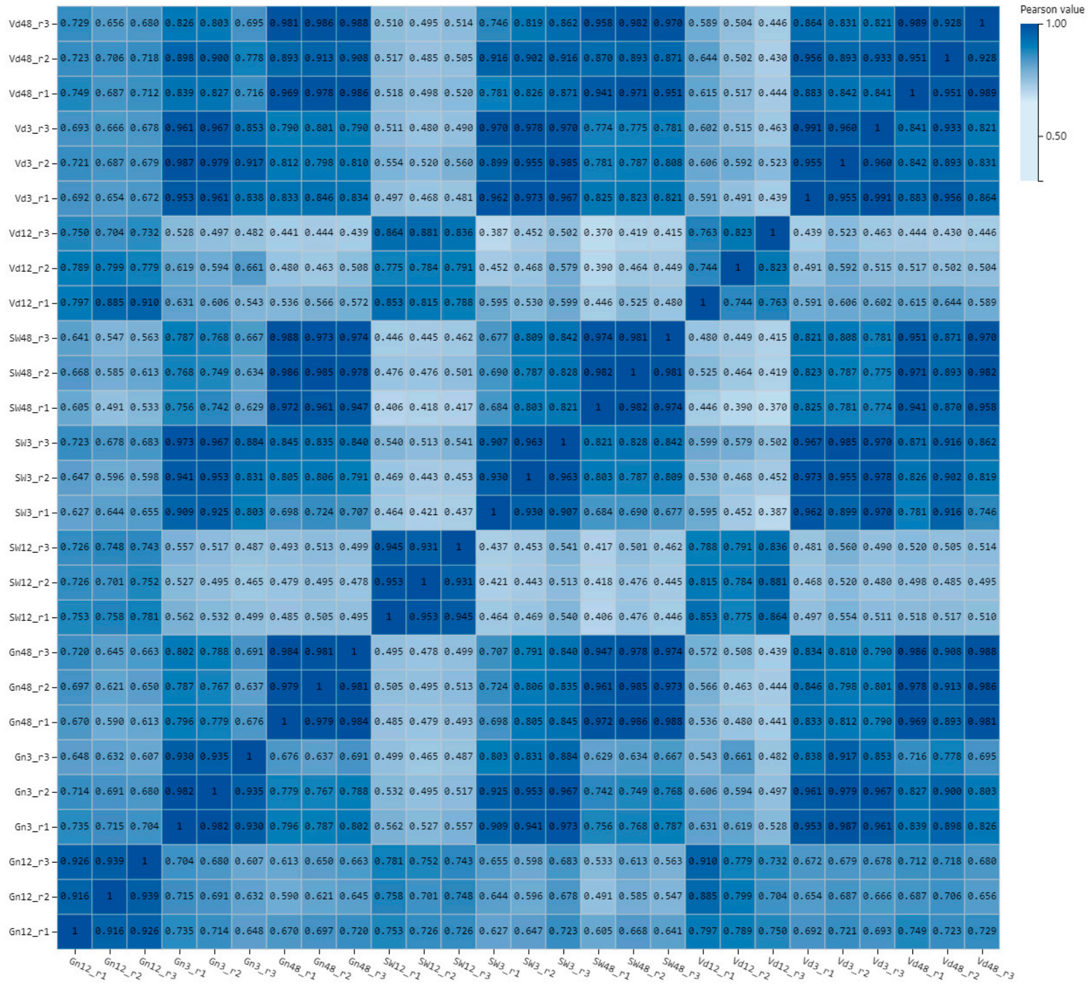
Pearson value
1.00
0.50
Vd48_r3
Vd48_r2
Vd48_r1
Vd3_r3
Vd3_r2
Vd3_r1
Vd12_r3
Vd12_r2
Vd12_r1
SW48_r3
SW48_r2
SW48_r1
SW3_r3
SW3_r2
SW3_r1
SW12_r3
SW12_r2
SW12_r1
Gn48_r3
Gn48_r2
Gn48_r1
Gn3_r3
Gn3_r2
Gn3_r1
Gn12_r3
Gn12_r2
Gn12_r1

Figure S2

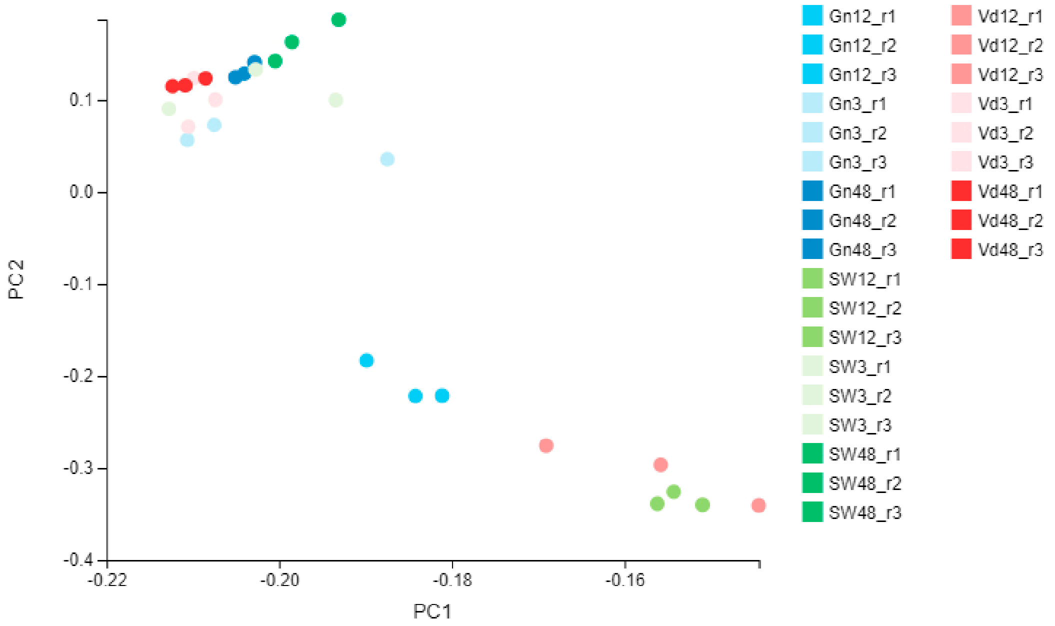
PC2
0.1
0.0
-0.1
-0.2
-0.3
-0.4
-0.22
-0.20
-0.18
-0.16
PC1
Gn12_r1
Gn12_r2
Gn12_r3
Gn3_r1
Gn3_r2
Gn3_r3
Gn48_r1
Gn48_r2
Gn48_r3
SW12_r1
SW12_r2
SW12_r3
SW3_r1
SW3_r2
SW3_r3
SW48_r1
SW48_r2
SW48_r3
Vd12_r1
Vd12_r2
Vd12_r3
Vd3_r1
Vd3_r2
Vd3_r3
Vd48_r1
Vd48_r2
Vd48_r3

Figure S3

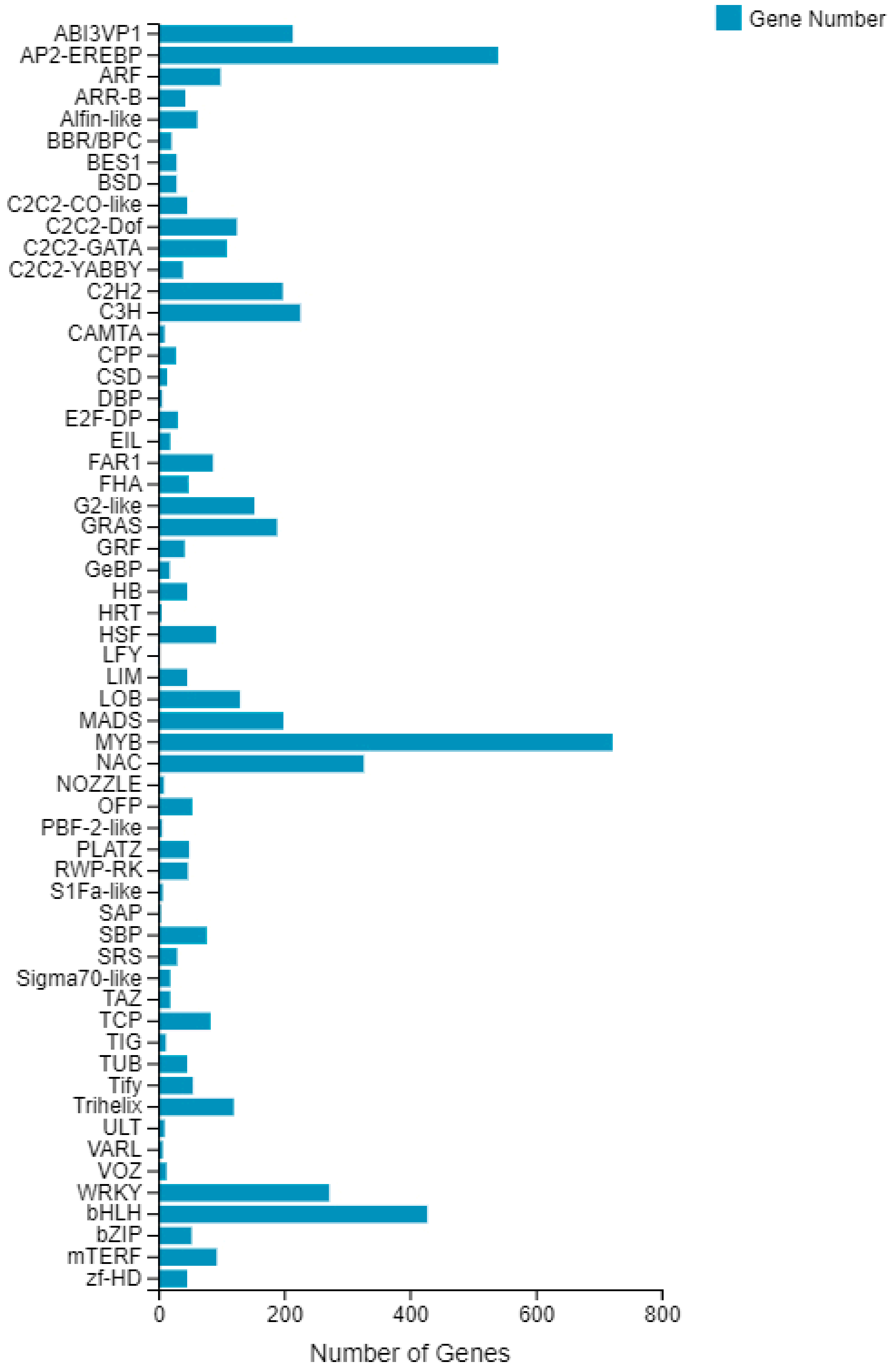
Gene Number
ABI3VP1
AP2-EREBP
ARF
ARR-B
Alfin-like
BBR/BPC
BES1
BSD
C2C2-CO-like
C2C2-Dof
C2C2-GATA
C2C2-YABBY
C2H2
C3H
CAMTA
CPP
CSD
DBP
E2F-DP
EIL
FAR1
FHA
G2-like
GRAS
GRF
GeBP
HB
HRT
HSF
LFY
LIM
LOB
MADS
MYB
NAC
NOZZLE
OFP
PBF-2-like
PLATZ
RWP-RK
S1Fa-like
SAP
SBP
SRS
Sigma70-like
TAZ
TCP
TIG
TUB
Tify
Trihelix
ULT
VARL
VOZ
WRKY
bHLH
bZIP
mTERF
zf-HD
0
200
400
600
800
Number of Genes

Figure S4

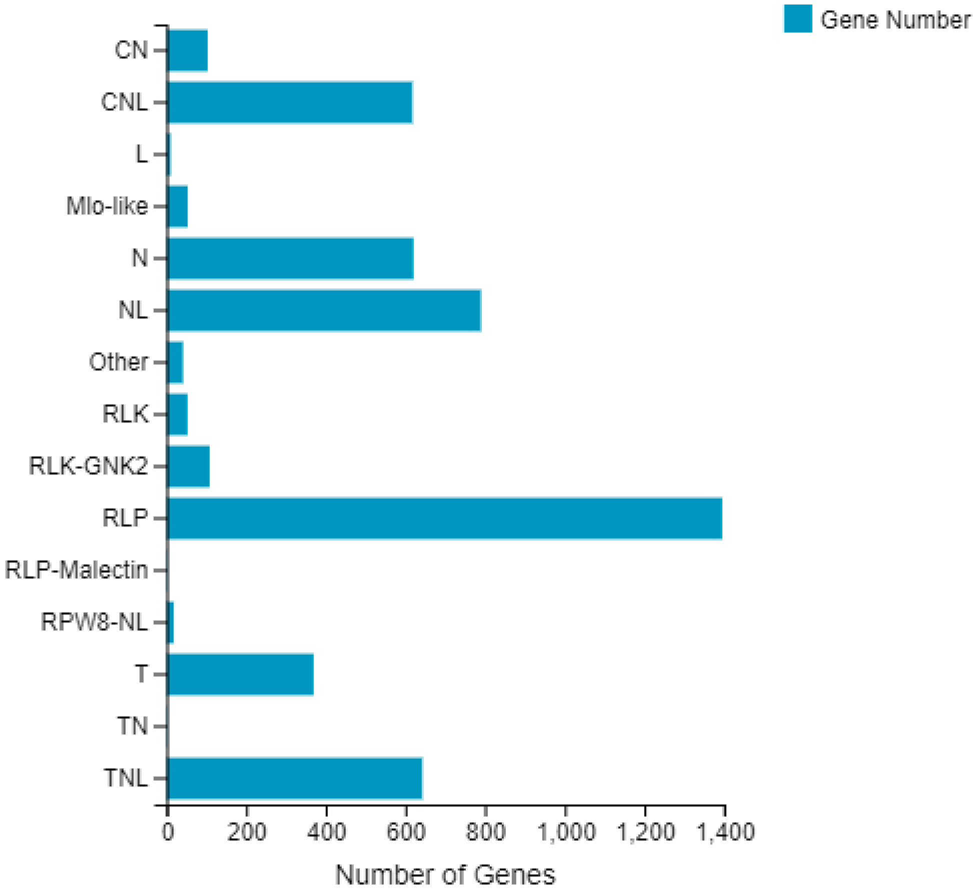

Gene Number
CN
CNL
L
Mlo-like
N
NL
Other
RLK
RLK-GNK2
RLP
RLP-Malectin
RPW8-NL
T
TN
TNL
0
200
400
600
800
1,000
1,200
1,400
Number of Genes

Figure S5

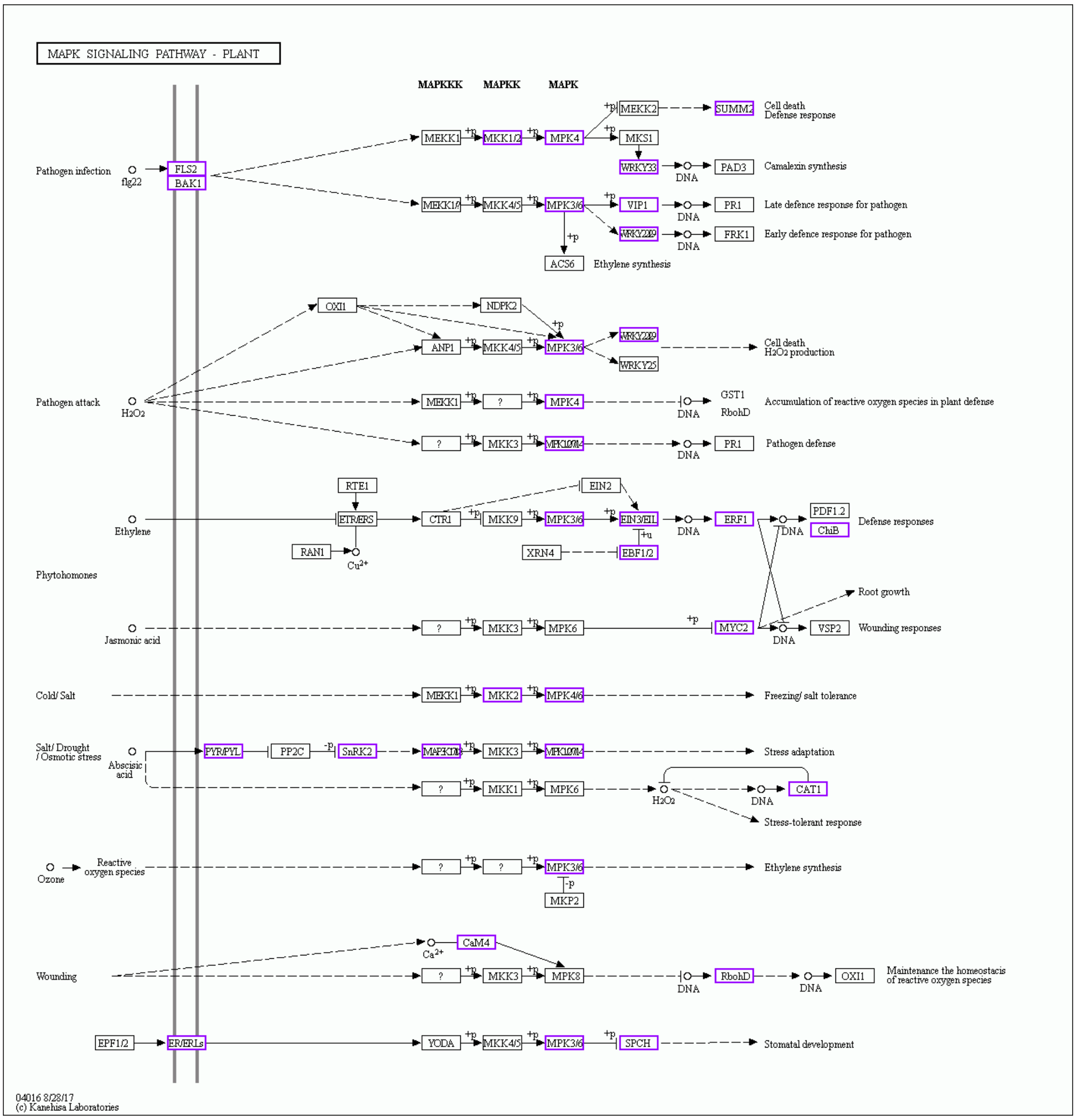

MAPK SIGNALING PATHWAY - PLANT
MAPKKK
MAPKK
MAPK
Pathogen infection
flg22
FLS2
BAK1
MEKK1
MKK1/2
MPK4
MEKK2
SUMM2
Cell death
Defense response
MKS1
WRKY33
DNA
PAD3
Camalexin synthesis
MEKK1/?
MKK4/5
MPK3/6
VIP1
PR1
Late defence response for pathogen
WRKY22/29
FRK1
Early defence response for pathogen
ACS6
Ethylene synthesis
OXI1
NDPK2
ANP1
WRKY22/29
WRKY25
Cell death
H2O2 production
Pathogen attack
H2O2
GST1
RbohD
Accumulation of reactive oxygen species in plant defense
MKK3
Pathogen defense
RTE1
EIN2
Ethylene
ETR/ERS
CTR1
MKK9
EIN3/EIL
ERF1
PDF1.2
ChiB
Defense responses
RAN1
Cu2+
XRN4
EBF1/2
Phytohomones
Root growth
Jasmonic acid
MPK6
MYC2
VSP2
Wounding responses
Cold/ Salt
MKK2
MPK4/6
Freezing/ salt tolerance
Salt/ Drought / Osmotic stress
Abscisic acid
PYR/PYL
PP2C
SnRK2
MKK3
Stress adaptation
MKK1
CAT1
Stress-tolerant response
Ozone
Reactive oxygen species
MKP2
Ethylene synthesis
Wounding
Ca2+
CaM4
MPK8
OXI1
Maintenance the homeostacis of reactive oxygen species
EPF1/2
ER/ERLs
YODA
SPCH
Stomatal development
04016 8/28/17
(c) Kanehisa Laboratories

Figure S6

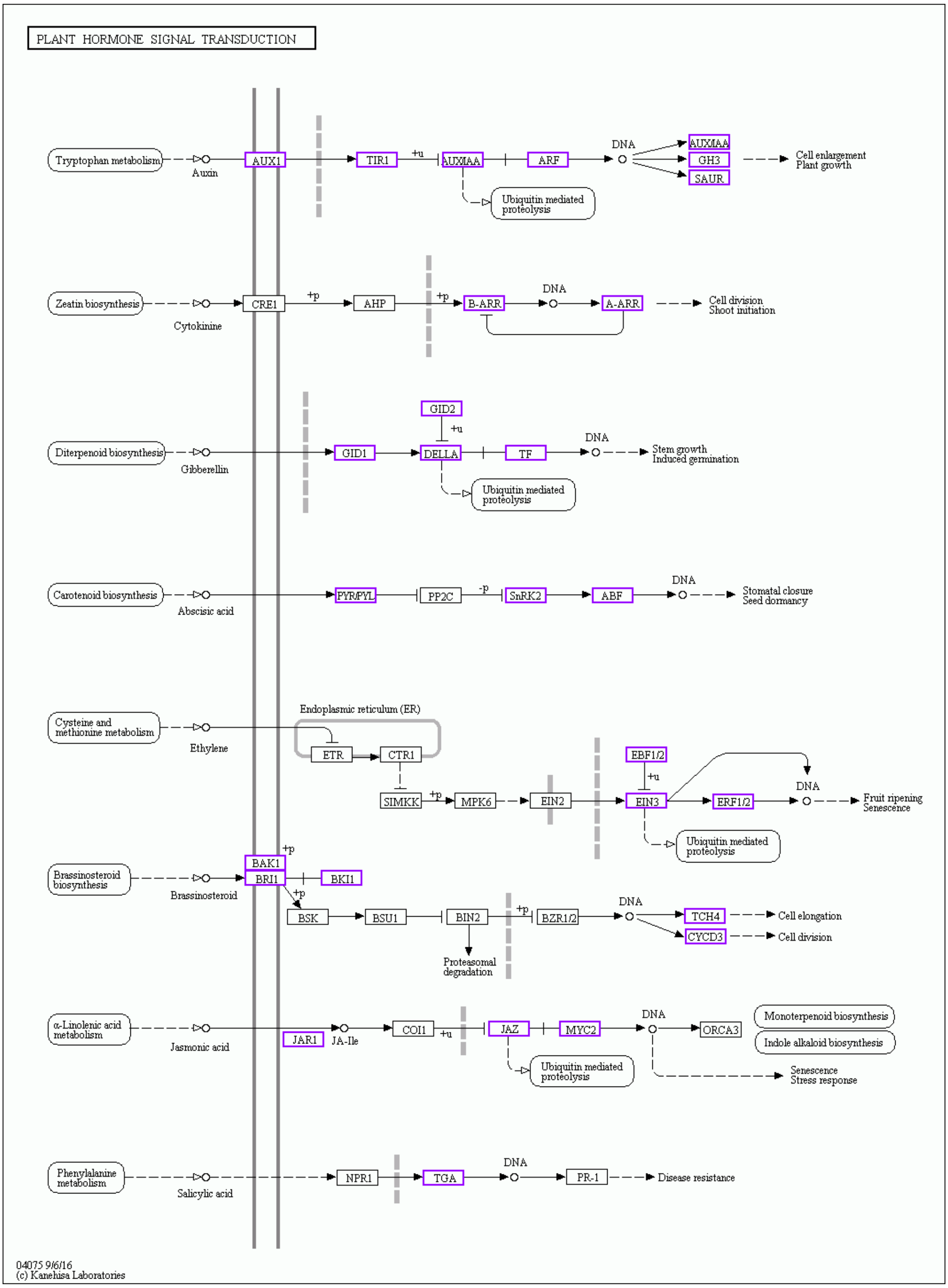
PLANT HORMONE SIGNAL TRANSDUCTION
Tryptophan metabolism
Auxin
AUX1
TIR1
+u
AUX/IAA
ARF
DNA
AUX/IAA
GH3
SAUR
Cell enlargement
Plant growth
Ubiquitin mediated proteolysis
Zeatin biosynthesis
Cytokinine
CRE1
+p
AHP
+p
B-ARR
DNA
A-ARR
Cell division
Shoot initiation
GID2
+u
Diterpenoid biosynthesis
Gibberellin
GID1
DELLA
TF
DNA
Stem growth
Induced germination
Ubiquitin mediated proteolysis
Carotenoid biosynthesis
Abscisic acid
PYR/PYL
PP2C
-p
SnRK2
ABF
DNA
Stomatal closure
Seed dormancy
Endoplasmic reticulum (ER)
Cysteine and methionine metabolism
Ethylene
ETR
CTR1
SIMKK
+p
MPK6
EIN2
EBF1/2
+u
EIN3
ERF1/2
DNA
Fruit ripening
Senescence
Ubiquitin mediated proteolysis
Brassinosteroid biosynthesis
Brassinosteroid
+p
BAK1
BRI1
BKI1
+p
BSK
BSU1
BIN2
+p
BZR1/2
DNA
TCH4
CYCD3
Cell elongation
Cell division
Proteasomal degradation
α-Linolenic acid metabolism
Jasmonic acid
JAR1
JA-Ile
COI1
+u
JAZ
MYC2
DNA
ORCA3
Monoterpenoid biosynthesis
Indole alkaloid biosynthesis
Senescence
Stress response
Ubiquitin mediated proteolysis
Phenylalanine metabolism
Salicylic acid
NPR1
TGA
DNA
PR-1
Disease resistance
04075 9/6/16
(c) Kanehisa Laboratories

Figure S7

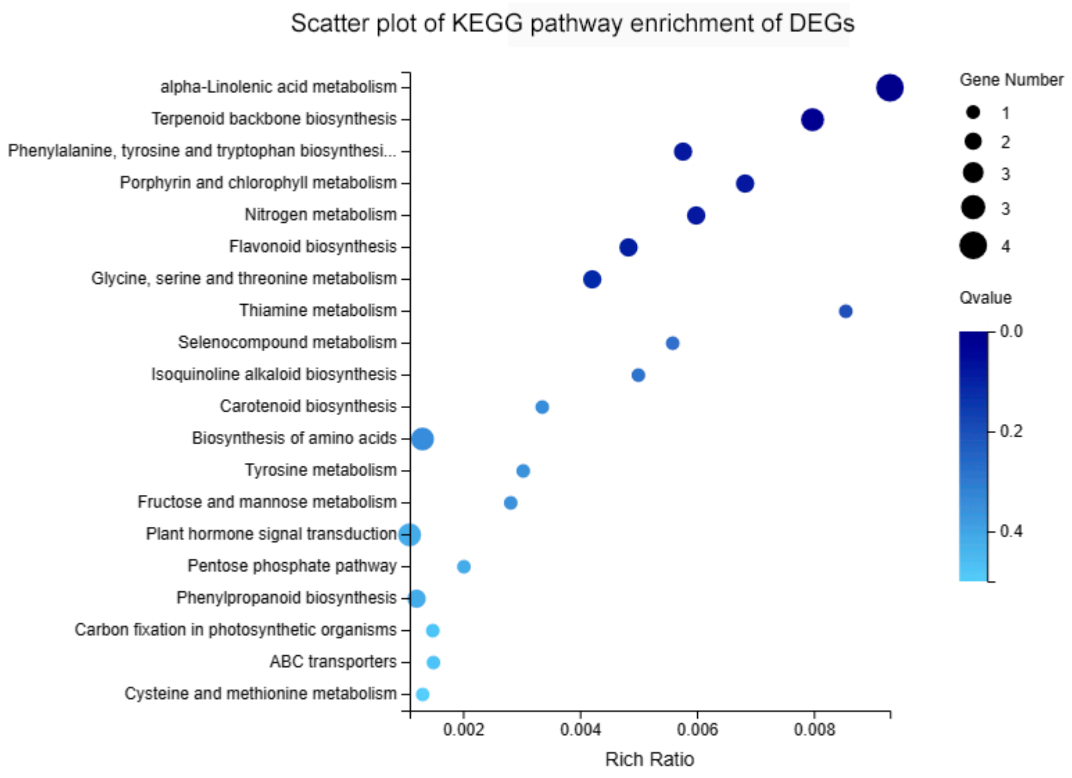

Scatter plot of KEGG pathway enrichment of DEGs
alpha-Linolenic acid metabolism
Terpenoid backbone biosynthesis
Phenylalanine, tyrosine and tryptophan biosynthesi...
Porphyrin and chlorophyll metabolism
Nitrogen metabolism
Flavonoid biosynthesis
Glycine, serine and threonine metabolism
Thiamine metabolism
Selenocompound metabolism
Isoquinoline alkaloid biosynthesis
Carotenoid biosynthesis
Biosynthesis of amino acids
Tyrosine metabolism
Fructose and mannose metabolism
Plant hormone signal transduction
Pentose phosphate pathway
Phenylpropanoid biosynthesis
Carbon fixation in photosynthetic organisms
ABC transporters
Cysteine and methionine metabolism
0.002
0.004
0.006
0.008
Rich Ratio
Gene Number
1
2
3
3
4
Qvalue
0.0
0.2
0.4

Figure S8

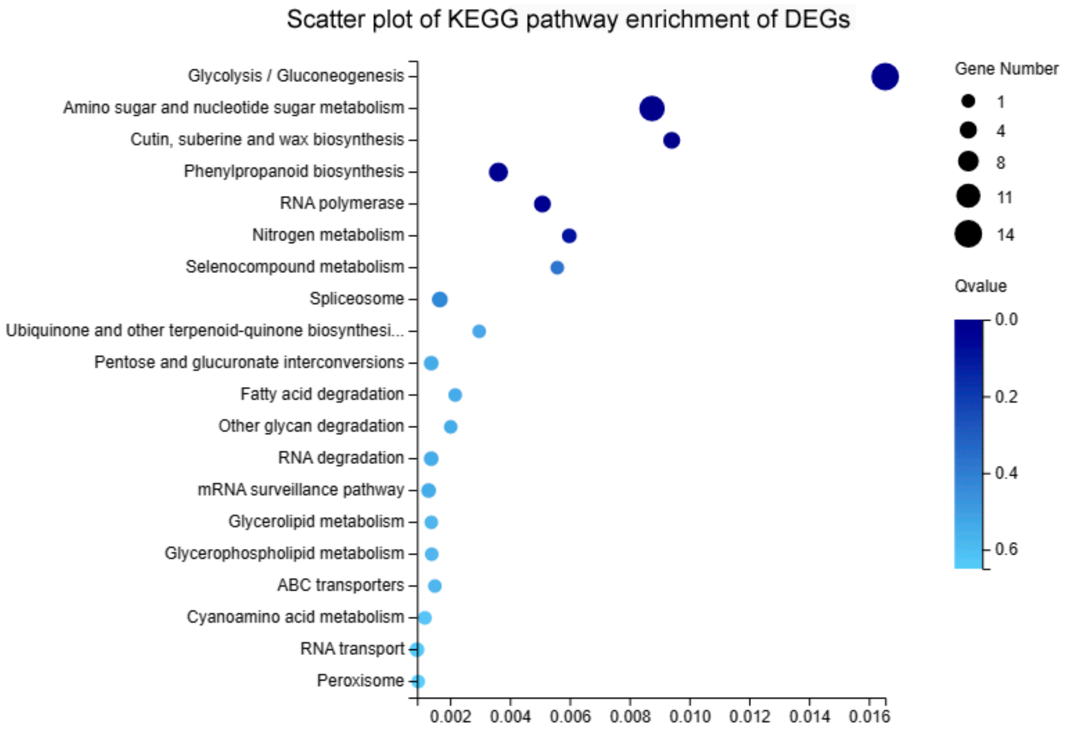
Scatter plot of KEGG pathway enrichment of DEGs
Glycolysis / Gluconeogenesis
Amino sugar and nucleotide sugar metabolism
Cutin, suberine and wax biosynthesis
Phenylpropanoid biosynthesis
RNA polymerase
Nitrogen metabolism
Selenocompound metabolism
Spliceosome
Ubiquinone and other terpenoid-quinone biosynthesi...
Pentose and glucuronate interconversions
Fatty acid degradation
Other glycan degradation
RNA degradation
mRNA surveillance pathway
Glycerolipid metabolism
Glycerophospholipid metabolism
ABC transporters
Cyanoamino acid metabolism
RNA transport
Peroxisome
0.002
0.004
0.006
0.008
0.010
0.012
0.014
0.016
Gene Number
1
4
8
11
14
Qvalue
0.0
0.2
0.4
0.6

Figure S9

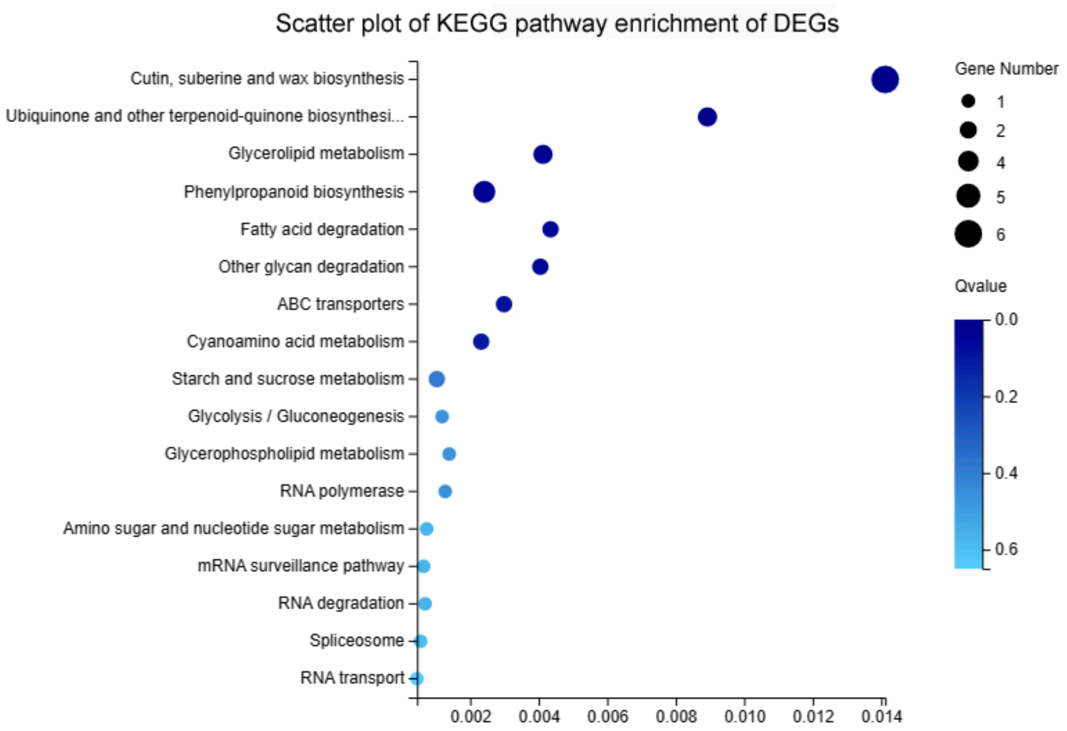

Scatter plot of KEGG pathway enrichment of DEGs
Cutin, suberine and wax biosynthesis
Ubiquinone and other terpenoid-quinone biosynthesi...
Glycerolipid metabolism
Phenylpropanoid biosynthesis
Fatty acid degradation
Other glycan degradation
ABC transporters
Cyanoamino acid metabolism
Starch and sucrose metabolism
Glycolysis / Gluconeogenesis
Glycerophospholipid metabolism
RNA polymerase
Amino sugar and nucleotide sugar metabolism
mRNA surveillance pathway
RNA degradation
Spliceosome
RNA transport
0.002
0.004
0.006
0.008
0.010
0.012
0.014
Gene Number
1
2
4
5
6
Qvalue
0.0
0.2
0.4
0.6

Figure S10

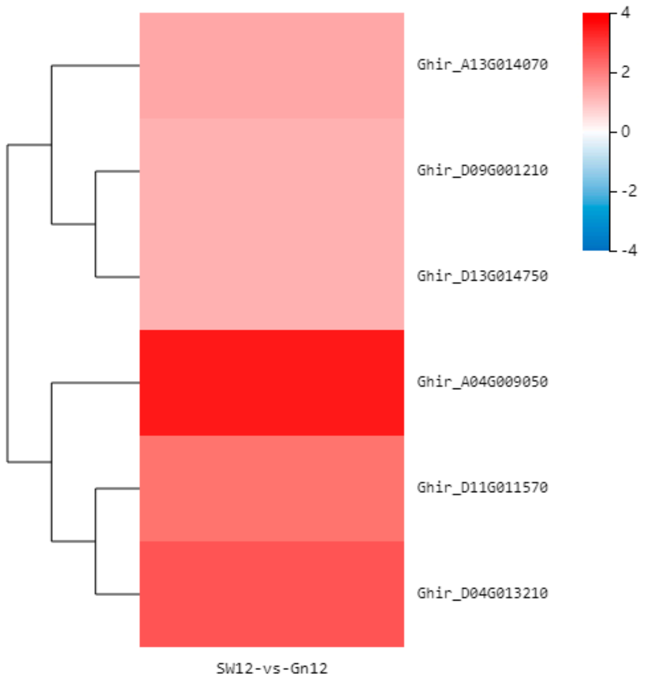
Ghir_A13G014070
Ghir_D09G001210
Ghir_D13G014750
Ghir_A04G009050
Ghir_D11G011570
Ghir_D04G013210
SW12-vs-Gn12
4
2
0
-2
-4

Figure S11

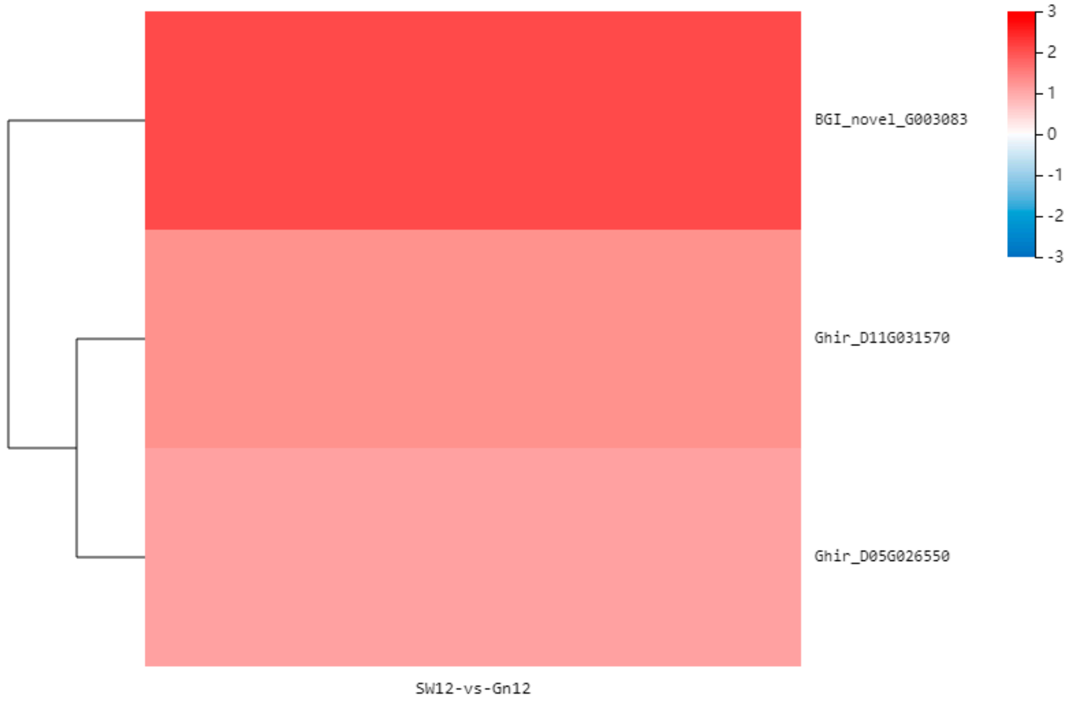

BGI_novel_G003083
Ghir_D11G031570
Ghir_D05G026550
SW12-vs-Gn12
3
2
1
0
-1
-2
-3

Figure S12

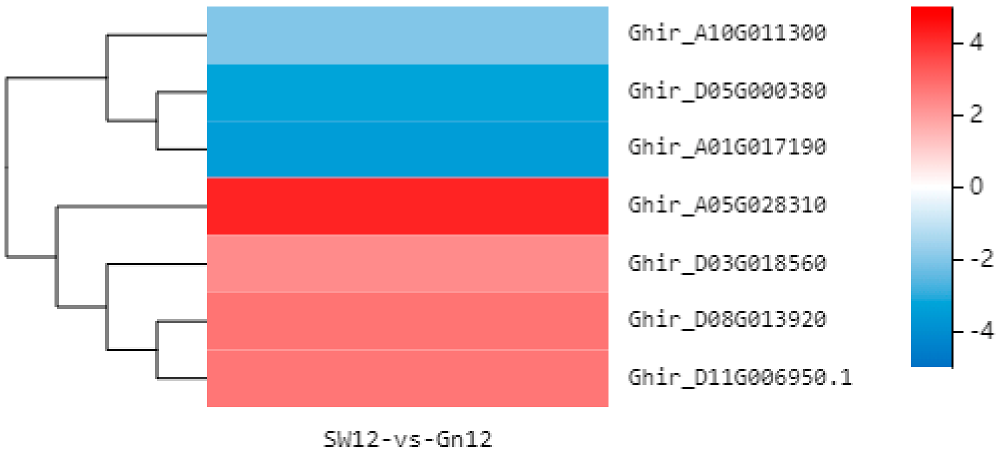
Ghir_A10G011300
Ghir_D05G000380
Ghir_A01G017190
Ghir_A05G028310
Ghir_D03G018560
Ghir_D08G013920
Ghir_D11G006950.1
4
2
0
-2
-4
SW12-vs-Gn12
